# Supplementary material for: Mef2C restrains microglial inflammatory response and is lost in brain ageing in an IFN-I-dependent manner
Source: Nat Commun. 2017 Sep 28;8:717. doi: 10.1038/s41467-017-00769-0 (PMC5620041; doi:10.1038/s41467-017-00769-0)
Supplement: Supplementary file 1 — Supplementary Information [file 41467_2017_769_MOESM1_ESM.pdf]

Description of Supplementary Files

File name: Supplementary Information
Description: Supplementary figures, supplementary table 1

File name: Supplementary Data 1
Description: Transcriptional profiling of microglia of young (3 month-old) and aged (22 month-old) mice.

File name: Supplementary Data 2
Description: Transcriptional profiling of microglia of untreated young mice (3 month-old) and aged (22 month-old) mice treated with anti-IFNAR or isotype control (IgG) antibodies 2, 7 or 14 days after the treatment.

File name: Supplementary Data 3
Description: Transcriptional profiling of microglia of young (3 month-old) mice infected with IFN-β-expressing (AAV-IFN-β) or empty (AAV-Ctrl) adeno-associated virus to their choroid plexi.

File name: Supplementary Data 4
Description: Transcriptional profiling of microglia of mic-IFNAR-CTRL and mic-IFNAR-KO mice infected with AAV-IFNβ or AAV-CTRL to their choroid plexi.

File name: Supplementary Data 5
Description: Transcriptional profiling of microglia of Mef2C-KO and Mef2C-CTRL mice.

File name: Peer Review File

a

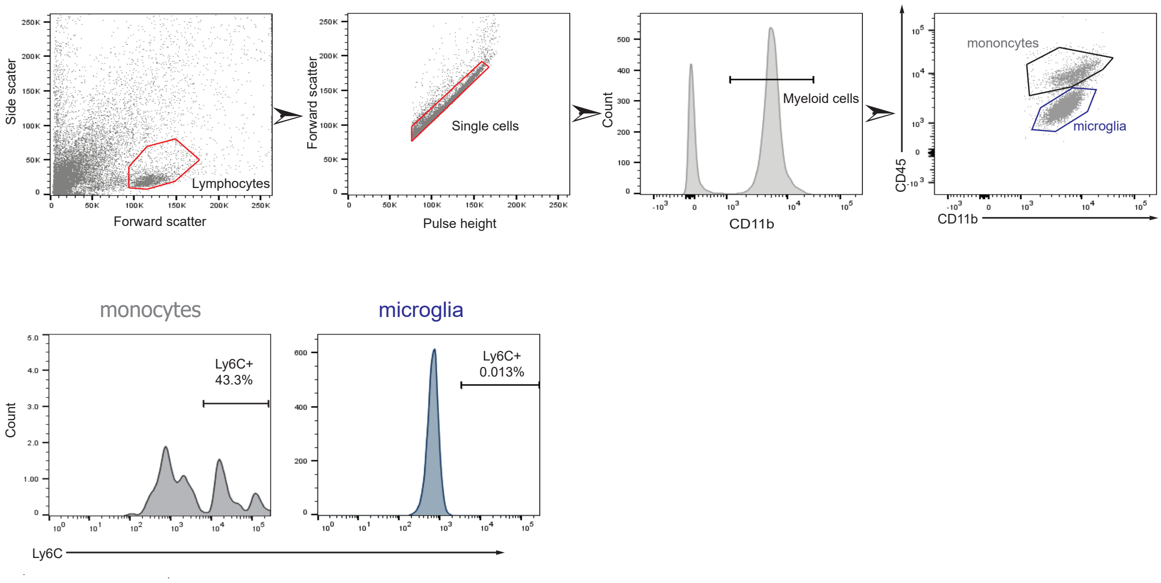

Supplementary Figure 1. Gating strategy used for sorting microglia (a). Gating strategy for microglia sorting according to the CD45 and CD11b staining levels. (b) Ly6C staining of the cells separated as microglia and monocytes.

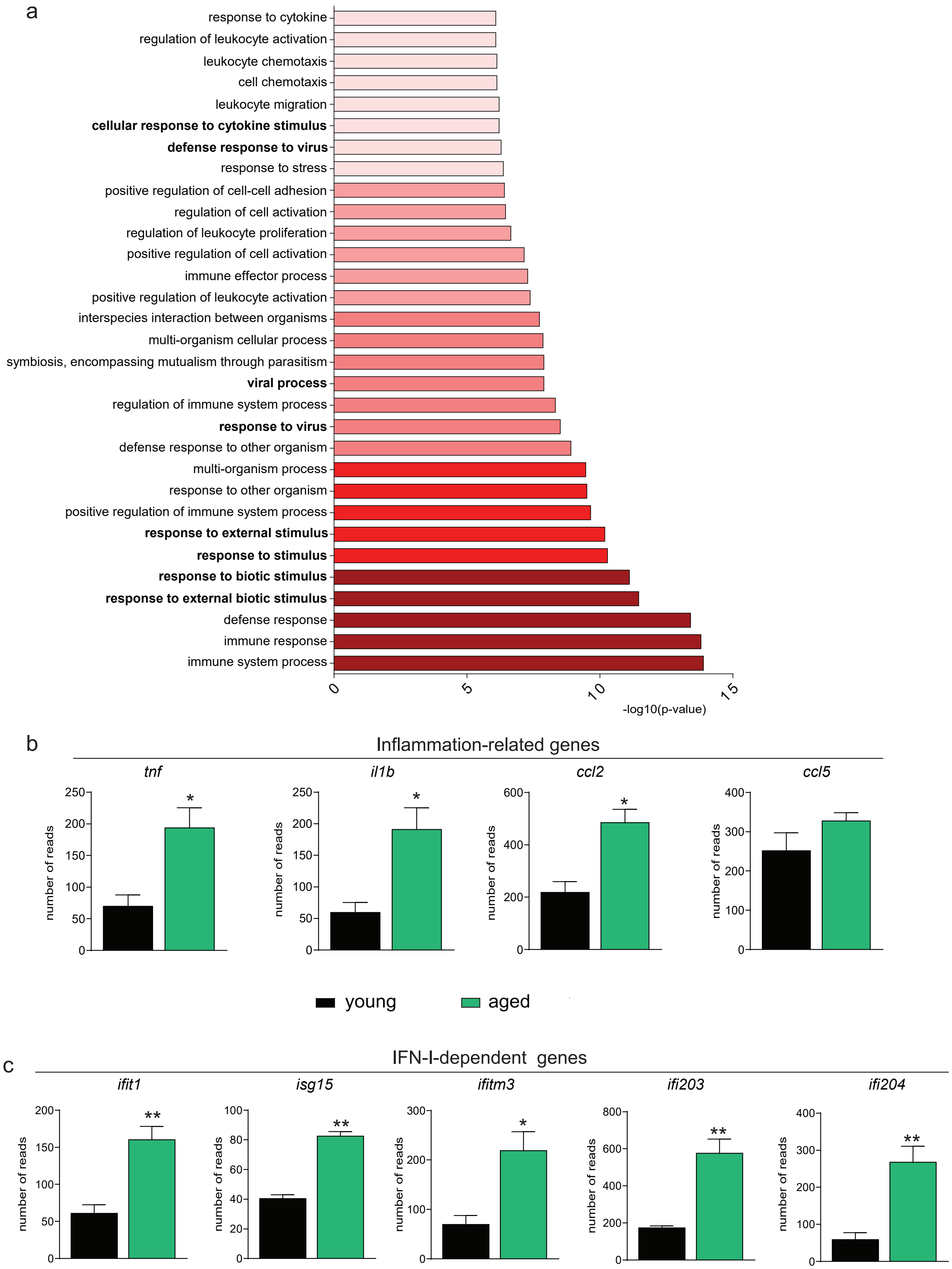

Supplementary Figure 2. Transcriptional profiling of aged microglia reveals immune- and virus-related responses. (a) RNA-seq results-based Gene Ontology (GO) enrichment analysis of genes significantly increased in microglia of aged mice, compared to young Bars represent -log10 (p-value) of enrichment computed according to the HG model. (b, c) mRNA expression levels, based on RNA-Seq, of inflammation-related *tnf*, *il1b*, *ccl2* and *ccl5* genes (b) and of IFN-I-dependent genes; *ifit1*, *isg15*, *ifitm3*, *ifi203*, *ifi204*; n=3, unpaired t-test, P * < 0.05, ** < 0.01.

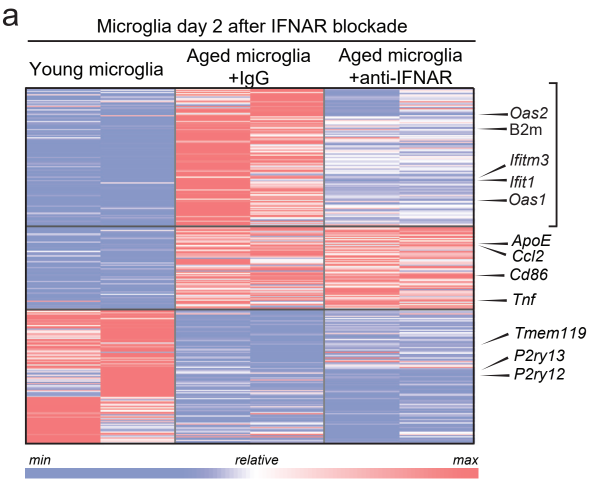

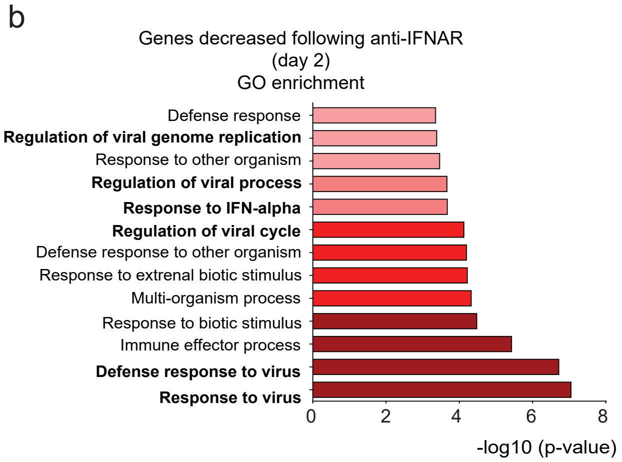

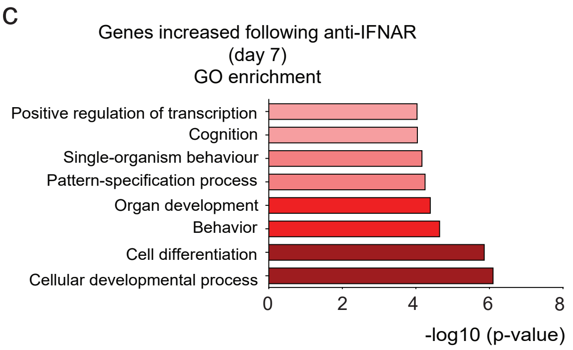

Supplementary Figure 3. Blocking IFN-I in aged brain microenvironment partly reverses aged microglial phenotype (a) Heat-map illustrating genes significantly changing between microglia of young mice and aged mice injected i.c.v. with IFNAR blocking antibody or IgG control two days following the treatment administration. (b,c) RNA-seq results-based GO enrichment analysis (b) of genes significantly reduced in microglia of anti-IFNAR-treated aged mice, compared to IgG control-injected mice, on day 2 following the antibody administration (c) of genes significantly increased in microglia of anti-IFNAR-treated aged mice compared to IgG control -injected mice on day 7 following the treatment. Bars represent -log10 (p-value) of enrichment computed according to the HG model.

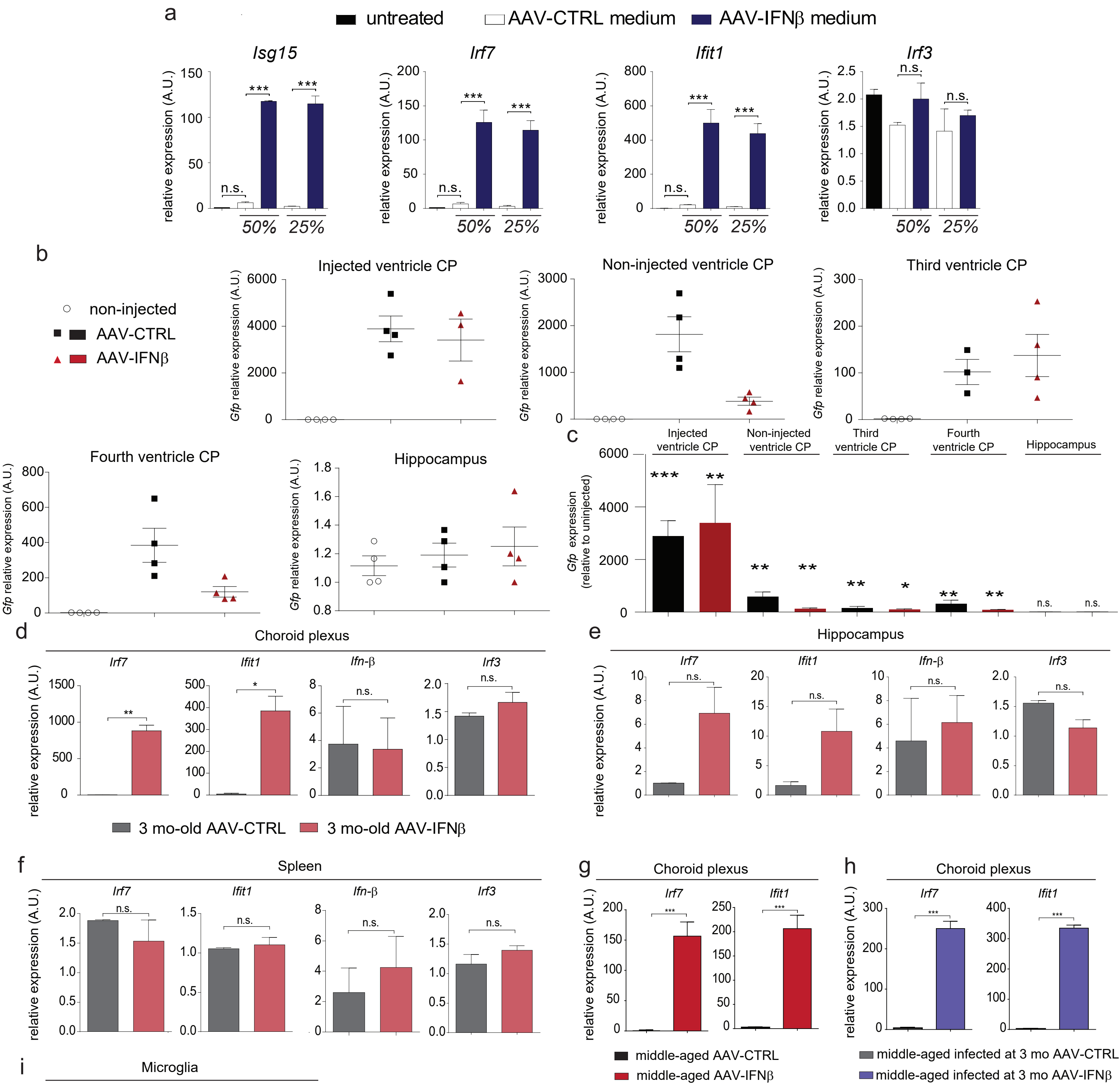

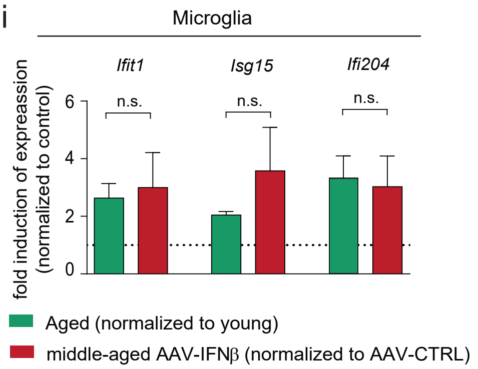

Supplementary Figure 4. Infection with AAV-IFNβ induces localized IFN-I response (a) Relative mRNA expression of IFN-I dependent *Isg15, Irf7* and *Ifit1* genes, and of *Irf3* (a gene involved in IFN-I response, but not directly regulated by IFNAR signaling), assessed by qPCR in choroid plexus epithelial cells in vitro which were untreated, or incubated with 25% or 50% of conditioned media of HEK293T cells used to prepare the AAV-IFNβ and AAV-Ctrl viruses. *Ppia* was used as reference gene. (n=3, one-way ANOVA with Newman-Kleus post-hoc test). (b) mRNA expression levels of GFP in choroid plexus of the injected (right) lateral ventricle, non-injected (left), contra-lateral ventricle, and third and fourth ventricles as well as right hemisphere hippocampus of AAV-CTRL or AAV-IFNβ-infected mice (n= 3-5). (c) mRNA expression levels of GFP in choroid plexus of the injected (right) lateral ventricle, non-injected (left) contra-lateral ventricle, and third and fourth ventricles as well as right hemi-sphere hippocampus of AAV-CTRL or AAV-IFNβ-infected mice. Results are normalized and displayed as fold change relative to non-injected (n=3-5, unpaired t-test for each one compared to non-injected). (d-f) relative expression levels of IFN-I dependent *Irf7, Ifit1*, endogenous *Ifnb* gene and of *Irf3*, determined by RT-qPCR, in the choroid plexus, hippocampus and spleen, 14 days after i.c.v. administration of the AAV-IFNβ or AAV-Ctrl (n=6, unpaired t-test) (g-h) Expression levels of IFN-I-dependent genes *Irf7* and *Ifit1*, determined by RT-qPCR, in the CP of middle-aged mice (12 month-old) mice 4 weeks (n=8-10, unpaired t-test) (g) or 7 months (n=5, unpaired t-test) (h) after the AAV-IFNβ or AAV-Ctrl administration. (i) Comparison of fold induction of expression of IFN-I-dependent *Ifit1, Isg15* and *Ifi204* genes in microglia of aged mice (normalized to young) and AAV-IFNβ mice (normalized to AAV-Ctrl), based on the RNA-Sequencing data (Supplementary Table 1, 3, 4), n=3-4, unpaired t-test) In all panels P * < 0.05, ** < 0.01, *** < 0.001.

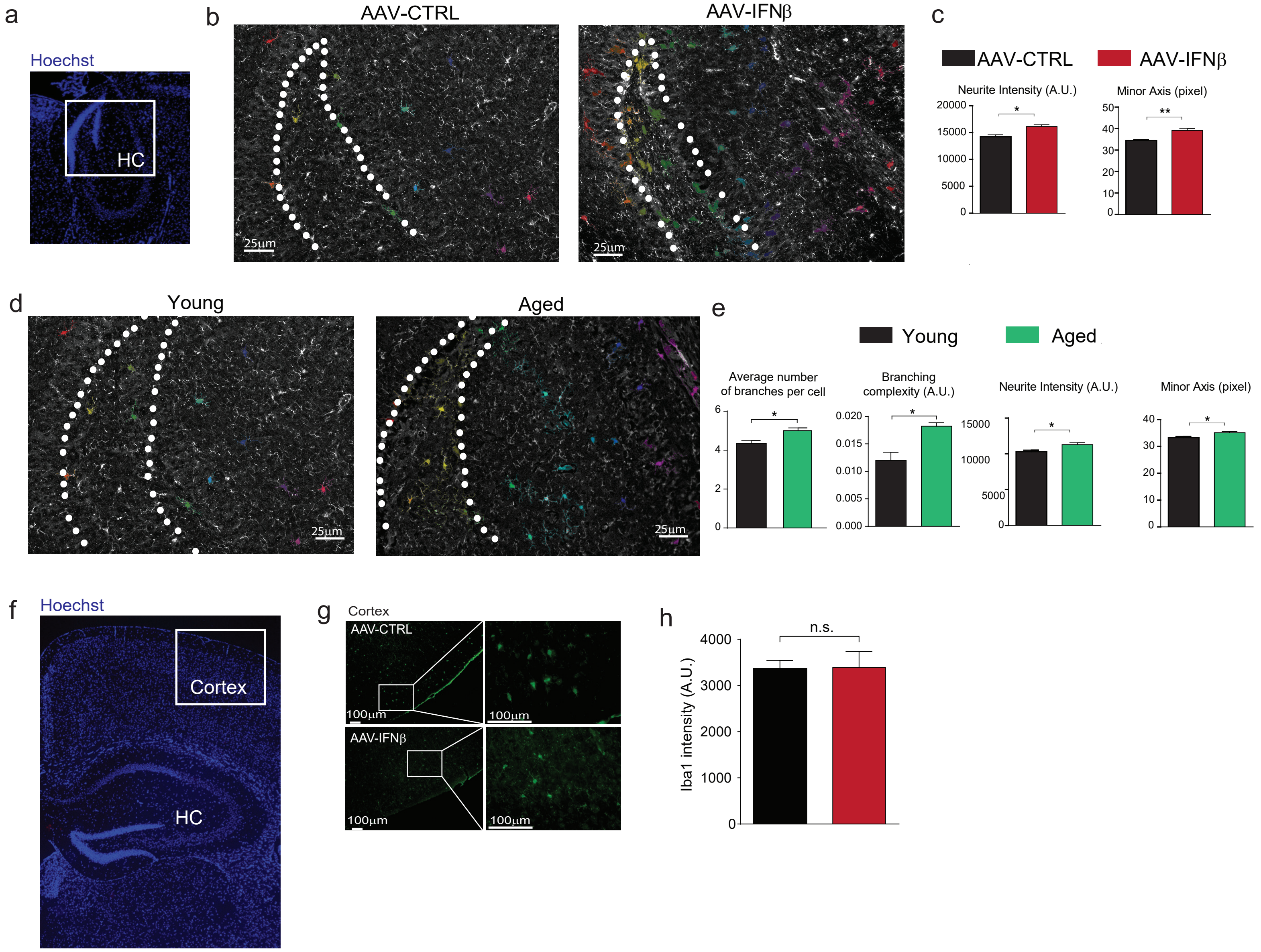

Supplementary Figure 5. Microglial morphology in young, aged and AAV-infected mice (a) Hoechst staining of murine brain section indicating the region of the hippocampus used for Iba1 intensity and morphology quantification (b) Representative images of microglial cell shape obtained by NeuroMath algorithm overlaid on the original images of Iba1-stained brain slices of AAV-Control and AAV-IFN-β-infected mice (c) Quantification of parameters obtained with NeuroMath algorithm by analyzing Iba1 staining in hippocampal slices of AAV-Control and AAV-IFN-β-infected mice. Data from all correctly recognized cell per mouse were averaged and compared (n=5). (d) Representative images of microglial cell shapes obtained by NeuroMath algorithm overlaid on the original images of Iba1-stained brain slices of young (3 months old) and aged (18-20 months old) mice. (e) Quantification of parameters obtained with NeuroMath algorithm by analyzing Iba1 staining in hippocampal slices of young and aged mice. Data from all correctly recognized cell per mouse were averaged and compared (n=5). (f) Hoechst staining of murine brain section indicating the region of the cortex used for Iba1 intensity quantification. (g) Representative images of Iba1 (green) microglial staining in the cortex of AAV-Control and AAV-IFN-β-infected mice. (h) Quantification of Iba1 intensity in the cortex of AAV-Control and AAV-IFN-β-infected mice. In all panels P * < 0.05, ** < 0.01, unpaired t test.

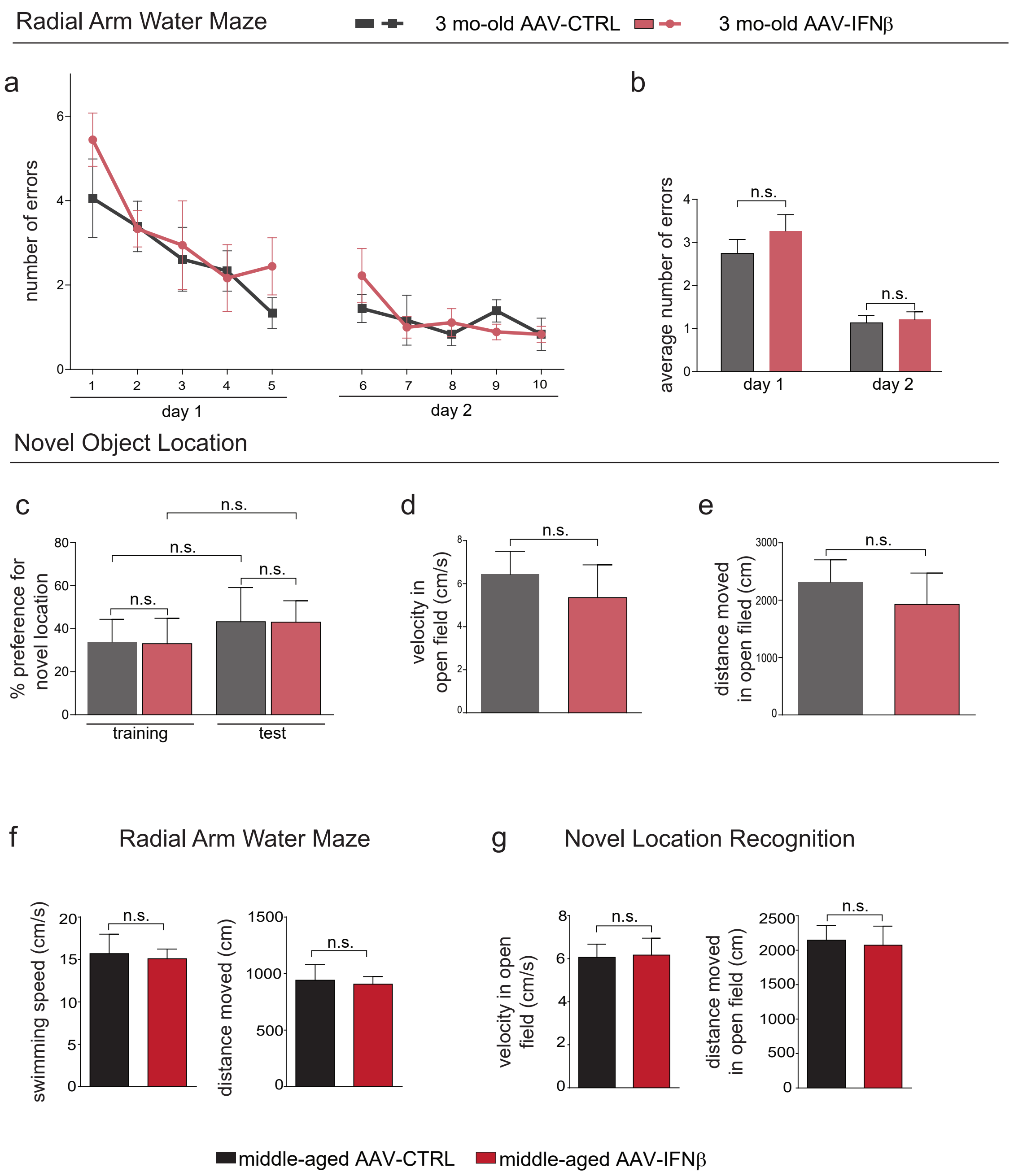

Supplementary Figure 6. IFN-I effect on cognitive ability in young and aged mice. (a-e) Hippocampal-dependent spatial learning and memoryof 3 months old mice 4 weeks after infection with AAV-IFN-β or AAV-CTRL virus to their choroid plexi. (a) Number of errors in RAWM task (two-way repeated- measures ANOVA with Bonferroni post-hoc test). (b) Averaged number of errors in RAWM task per group per day (paired t test). (c-e) Performance in Novel Location Recognition task (one-way ANOVAwith Newman-Kleus post-hoc test). (c) Percentage of time spent exploring the object in new location (one-way ANOVA with Newman-Kleus post-hoc test). (d) Average velocity and (e) distance moved in the arenaduring a single trial (unpaired t test). (f) Average swimming speed and distance moved over 1 minute in RAWM pool in 12 month-old mice infected with AAV-IFNβ or AAV-Ctrl 14 days after administration of the viruses (unpaired t test). (g) Average velocity and distance moved over 1 minute in open field in 12 month-old mice infected with AAV-IFNβ or AAV-Ctrl 14 days after administration of the viruses (unpaired t test). In all panels n=8-10 per group.

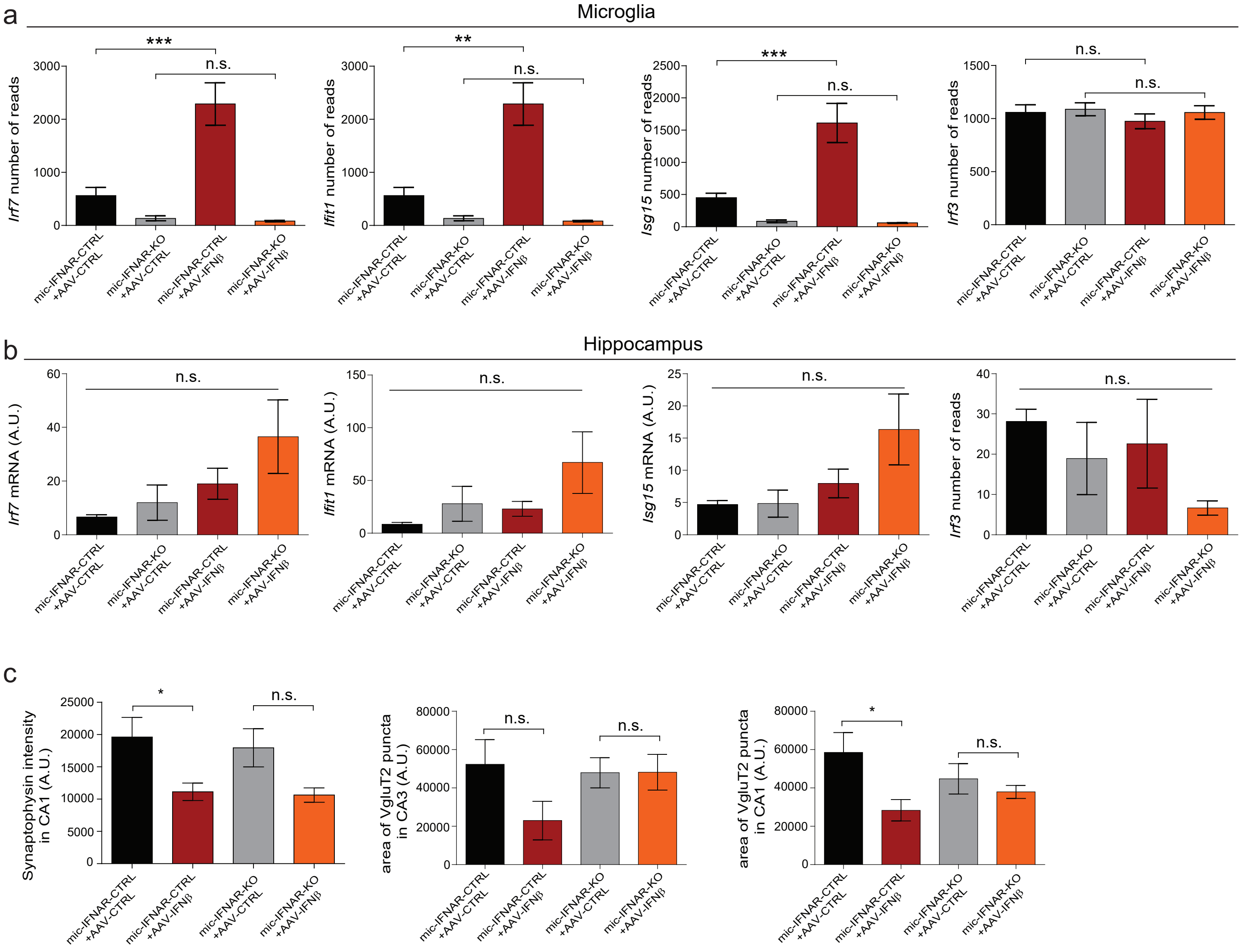

Supplementary Figure 7. Effects of AAV-mediated IFNβ overexpression are abrogated in mic-IFNAR-KO mice. (a) Number of transcript reads, obtained by RNA-Sequencing, of *Irf7, Ifit1, Isg15* and , and of *Irf3*, a gene involved in IFN-I response, but not directly regulated by IFNAR signaling in microglia of middle-aged micIFNAR-CTRL and micIFNAR-KO mice infected with AAV-CTRL and AAV-IFN-β (One-way ANOVA with Newman-Kleus post-hoc test; n=4-5 per group). (b) Relative mRNA expression levels, obtained by qPCR, of *Irf7, Ifit1, Isg15* and *Irf3* in hippocampi of the i.c.v. injected hemisphere of middle-aged micIFNAR-CTRL and micIFNAR-KO mice infected with AAV-CTRL and AAV-IFN-β. Gene expression was normalized relative to the housekeeping gene *Hprt*. (One-way ANOVA with Newman-Kleus post-hoc test; n=4-5 per group). (c) Quantification of presynaptic marker, synaptophysin intensity in CA1 and VgluT2+ puncta in CA3 and CA1 of the hippocampus of middle-aged mic-IFNAR-CTRL and mic-IFNAR-KO mice infected with AAV-CTRL and AAV-IFNβ (unpaired t-test between AAV-CTRL and AAV-IFNβ-infected mice, n=4-5 per group). In all panels P *** < 0.001, P ** < 0.01, P * < 0.05.

a

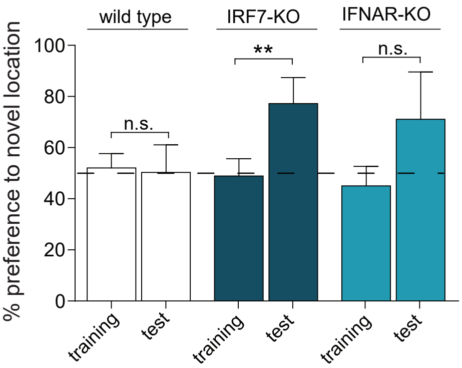

Supplementary Figure 8. Mice lacking IFN-I signaling are protected from cognitive loss in aging.
(a) Performance of aged (22 mo-old) wild type, IRF-7-KO and IFNAR-KO mice in Novel Location Recognition task for assessment of hippocampal memory. P ** < 0.01, unpaired t test, n=6-7 per group.

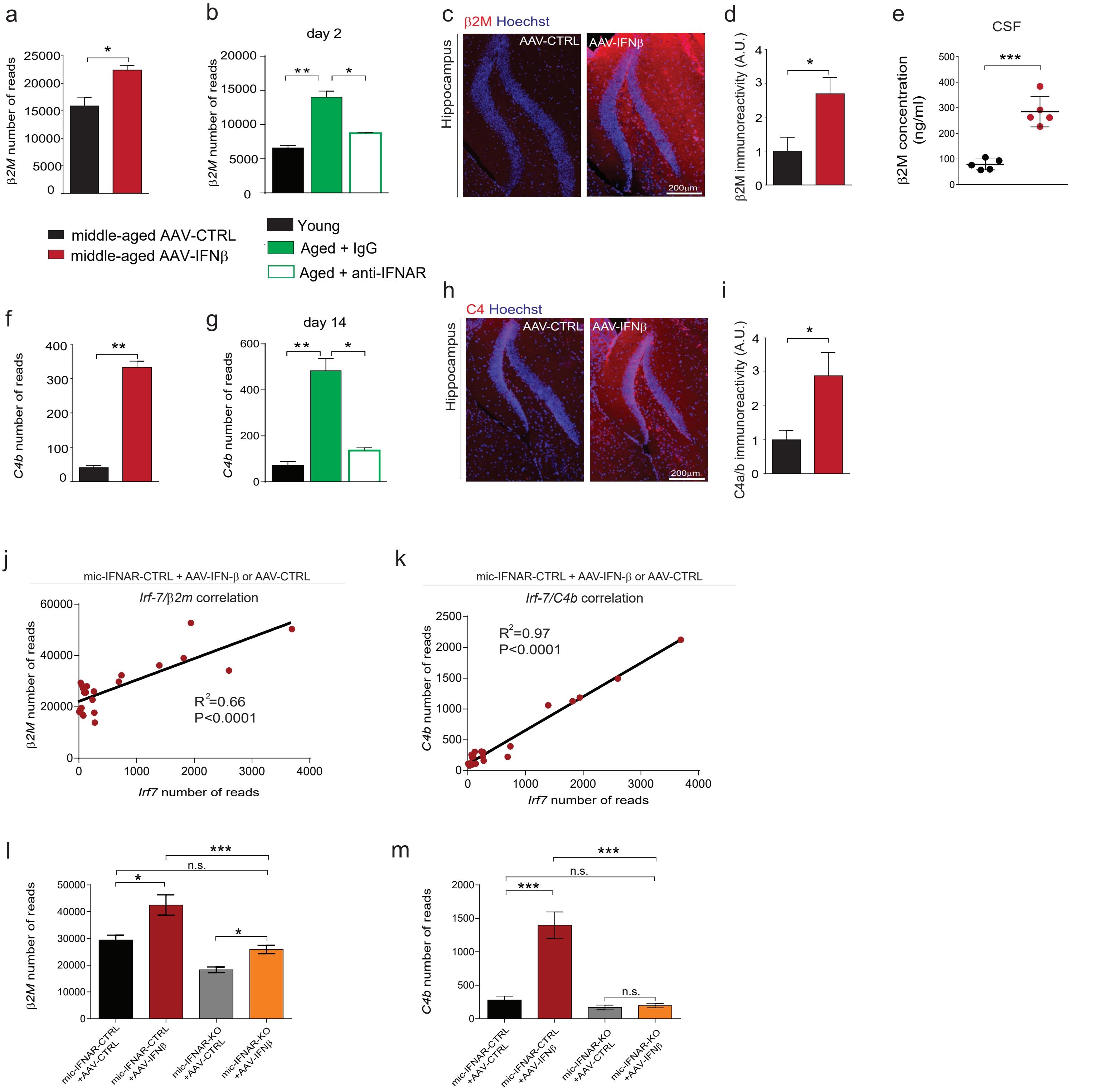

Supplementary Figure 9. AAV-induced IFN-β overexpression induces B2M and C4 expression in microglia. (a) mRNA expression levels (number of reads) of *β2M* in microglia of middle-aged IFN-I-overexpressing and control mice obtained by RNA sequencing (n=3, unpaired t test). (b) mRNA expression levels (number of reads), obtained by RNA sequencing, of *β2M* in microglia of young and aged mice i.c.v. injected with anti-IFNAR antibody of IgG control antibody two days following the injection (n=3, one-way ANOVA with Newman-Kleus post-hoc test). (c) Representative micrographs of β2M staining (red) in microglia of mice infected with IFN-I-overexpressing or control AAV (nuclear Hoechst staining in blue). (d) Quantification of β2M staining intensity in middle-aged IFN-I-overexpressing and control mice (n=5, unpaired t test). (e) Concentration of soluble β2M levels in cerebrospinal fluid collected from middle-aged IFN-I-overexpressing and control mice (n=5, unpaired t test). (f) mRNA expression levels (number of reads) of *C4b* in microglia of middle-aged IFN-I-overexpressing and AAV-control mice obtained by RNA sequencing (n=3, unpaired t test). (g) mRNA expression levels (number of reads), obtained by RNA sequencing, of *C4b* in microglia of young and aged mice i.c.v. injected with anti-IFNAR antibody of IgG control antibody 14 days following the injection (n=3, one-way ANOVA with Newman-Kleus post-hoc test). (h) Representative micrographs of C4 staining (red) in microglia of mice infected with IFN-I-overexpressing or control AAV (nuclear Hoechst staining in blue). (i) Quantification of C4 staining intensity in middle-aged IFN-I-overexpressing and AAV-control mice (n=5, unpaired t test). (j-k) Correlation analysis between expression levels of IFN-I-dependent Irf7 and β2m (r=0.8152) (j) and *C4b* (r=0.9852) (k) reveals positive relationship in microglia of micIFNAR-CTRL infected with AAV-CTRL and AAV-IFN-β (n=18, P<0.0001). (l-m) mRNA expression levels (number of reads) of *β2M* (l) and *C4b* (m) obtained by RNA sequencing in microglia of middle-aged micIFNAR-CTRL and micIFNAR-KO mice infected with AAV-CTRL and AAV-IFN-β suggests that β2m and *C4b* expression in microglia are direct effects of microglial IFNAR stimulation (n=3-5, one-way ANOVA with Newman-Kleus post-hoc test). In all panels P *** < 0.001, P ** < 0.01, P * < 0.05.

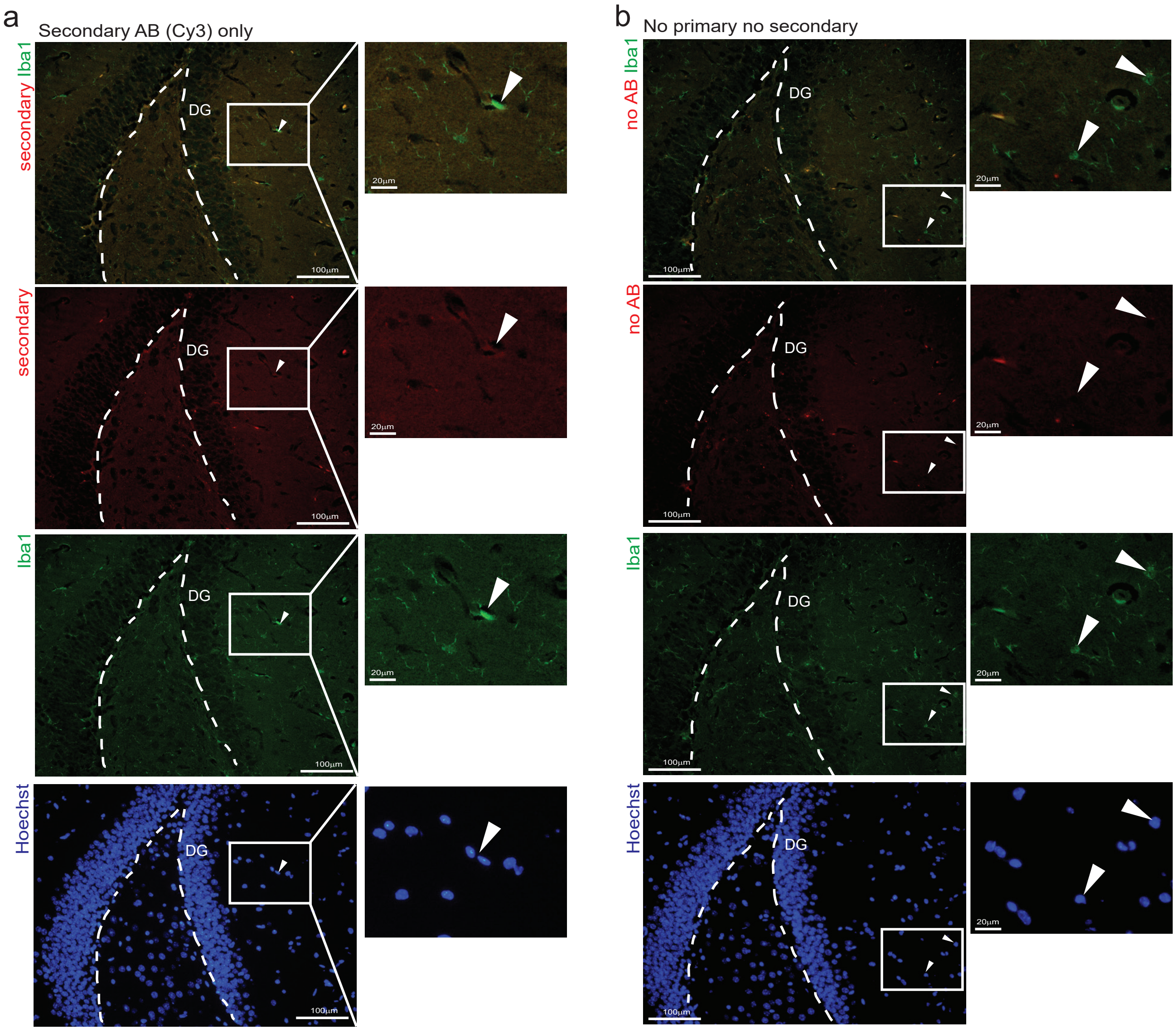

Supplementary Figure 10. Microglial Mef2C staining controls. (a-b) representative images showing control staining for immunohistochemical detection of Mef2C. (a) Staining for Iba-1 (green) and Hoechst nuclear staining (blue); anti-Mef2C antibody was omitted from the protocol and secondary Cy3-conjugated anti- mouse antibody was used to exclude non-specific binding of this antibody (b) Staining for Iba-1 (green) and Hoechst nuclear staining (blue); anti-Mef2C antibody and Cy3-conjugated anti-mouse were omitted from the protocol to exclude non-specific Mef2C/Cy3 signal.

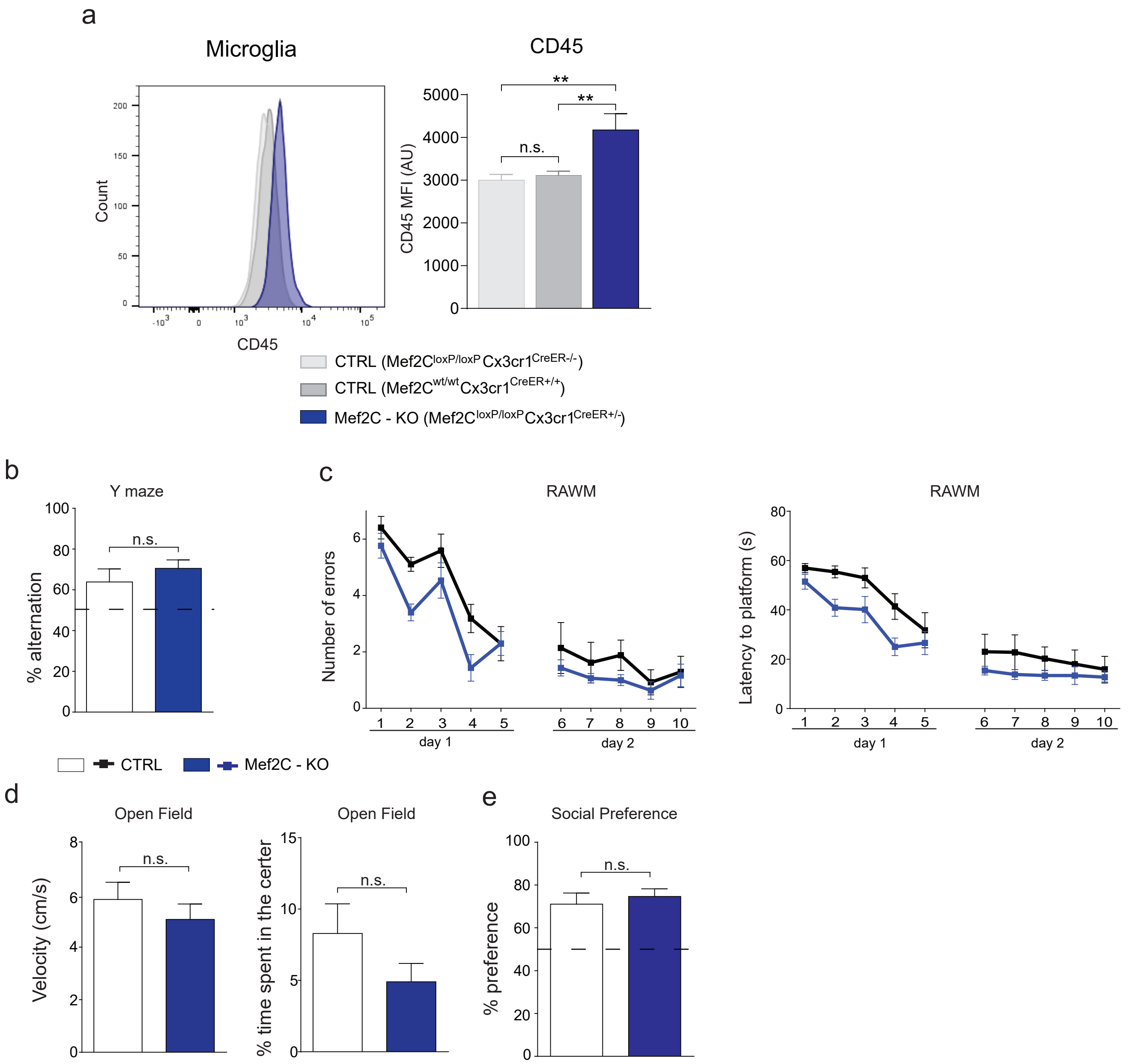

Supplementary Figure 11. Mef2C-KO mice show increased microglial CD45, but no behavioral phenotype (a) Representative FACS histograms and mean fluorescent intensity of CD45 staining in microglia of Mef2C-KO (Mef2CloxP/loxP::Cx3cr1ERT2-Cre+/-) and two types of Mef2C-CTRL mice (Mef2Cwt/wt::Cx3cr1ERT2-Cre+/- and Mef2CloxP/loxP::Cx3cr1ERT2-Cre-/-). P**<0.01; one-way ANOVA with Newman-Kleus post-hoc test. (b-e) Performance of Mef2C-KO and CTRL mice in (a) Y maze (unpaired t-test) (b) RAWM (two-way repeated-measures ANOVA with Bonferroni post-hoc test) (c) open filed exploration (unpaired t-test) (d) social preference test (unpaired t-test). In all panels n=9-10 per group.

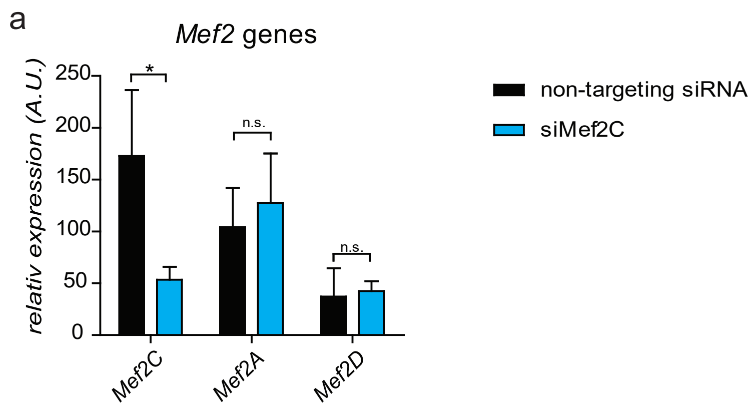

Supplementary Figure 12. siRNA-mediated downregulation of Mef2C in prmary cultures of microglia
(a) Number of reads for mRNA transcripts of Mef2C, Mef2A and Mef2D obtained in RNA-seq of siMef2C- and non-targeting siRNA-transfected microglia suggest specific loss of Mef2C.

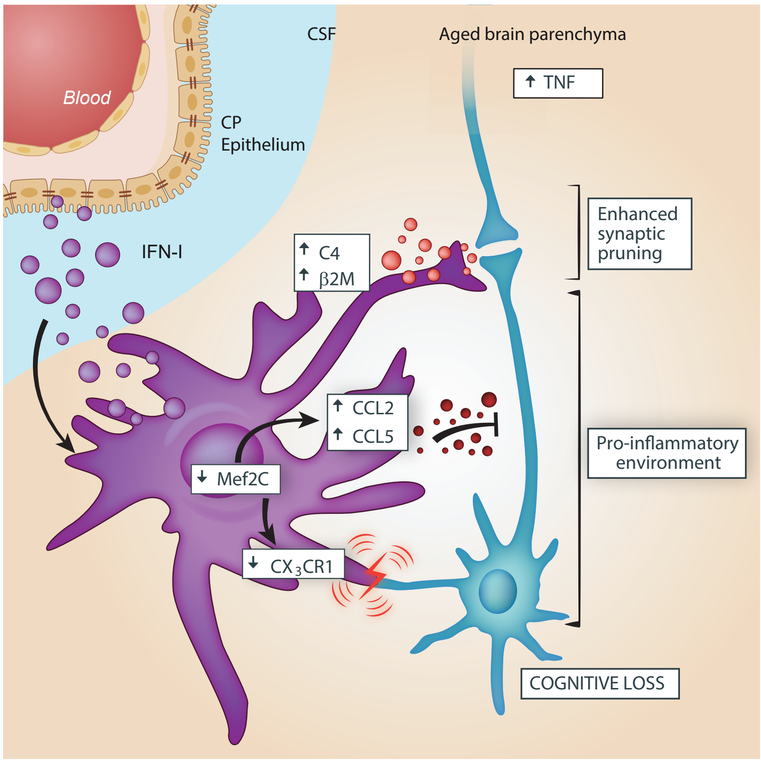

Supplementary Figure 13. Proposed model of IFN-I dependent activities of microglia in aging

Target	Forward	Reverse
ppia	5'-AGCATACAGGTCCTGGCATCTTGT-3'	5'-CAAAGACCACATGCTTGCCATCCA-3'
hprt	5’-GCTATAAATTCTTTGCTGACCTGCTG-3’	5’-AATTACTTTTATGTCCCCTGTTGACTGG-3’
gfp	5'-ACGTAAACGGCCACAAGTTC-3'	5'-AAGTCGTGCTGCTTCATGTG-3'
ifit1	5’-CTTTACAGCAACCATGGGAGAG-3’	5’-TCCATGTGAAGTGACATCTCAG-3’
irf7	5’-GCACTTTCTTCCGAGAACTGG-3	5’-CCTGCTGACAAGTCTTGCC-3’
b2m	5’-TGGTCTTTCTATATCCTGGCTC-3’	5’-TCTCGATCCCAGTAGACGG-3’
ifn-b	5’-CTGGCTTCCATCATGAACAA-3’	5’-AGAGGGCTGTGGTGGAGAA-3’
isg15	5’-CAATGGCCTGGGACCTAA-3’	5’-CGGCACACCAATCTTCTG-3’
irf3	5’-CTCTGTGGTTCTGCATGGG -3’	5’-TGTAGGAACAACCTTGACCA-3’
ccl5	5’-GTGCTCCAATCTTGCAGTCGTGTT-3’	5’-ACTTCTTCTCTGGGTTGGCACACA-3’
ccl2	5’-GGTCCCTGTCATGCTTCTG-3’	5’-GTTAACTGCATCTGGCTGAG-3’
il-1b	5’-CTTTGAAGAAGAGCCCATCC-3’	5’-GTTGTTCATCTCGGAGCCT-3’
tnf	5’-CCACGTCGTAGCAAACCAC-3’	5’-GTCTTTGAGATCCATGCCGT-3’

Supplementary Table 1. Sequeneces of the primers used in the study
