# Supplementary material for: Mef2C restrains microglial inflammatory response and is lost in brain ageing in an IFN-I-dependent manner
Source: Nat Commun. 2017 Sep 28;8:717. doi: 10.1038/s41467-017-00769-0 (PMC5620041; doi:10.1038/s41467-017-00769-0)
Supplement: Supplementary file 3 — Description of Additional Supplementary Files [file 41467_2017_769_MOESM3_ESM.pdf]

Description of Additional Supplementary Files

File name: Supplementary Data 1
Description: Transcriptional profiling of microglia of young (3 month-old) and aged (22 month-old) mice.

File name: Supplementary Data 2
Description: Transcriptional profiling of microglia of untreated young mice (3 month-old) and aged (22 month-old) mice treated with anti-IFNAR or isotype control (IgG) antibodies 2, 7 or 14 days after the treatment.

File name: Supplementary Data 3
Description: Transcriptional profiling of microglia of young (3 month-old) mice infected with IFN-β- expressing (AAV-IFN-β) or empty (AAV-Ctrl) adeno-associated virus to their choroid plexi.

File name: Supplementary Data 4
Description: Transcriptional profiling of microglia of mic-IFNAR-CTRL and mic-IFNAR-KO mice infected with AAV-IFNβ or AAV-CTRL to their choroid plexi.

File name: Supplementary Data 5
Description: Transcriptional profiling of microglia of Mef2C-KO and Mef2C-CTRL mice.
